# 

**Published:** 1890-04

**Authors:** 


					﻿Vol. X.
'APRIL, 1890.
No. 4;
THE
HOMEOPATHIC pHY^lClAfl,
A. Monthly Journal of Homoeopathic Materia Medica
and Clinical Medicine.
“ He is a freeman whom truth makes free, and all are slaves beside.”
“ Seek the Truth: come whence it may, cost -what it will.”
EDITED AND PUBLISHED 6y
WALTER M. JAMES, M. E>.,
AND
George H. Clark, ni. d.
ASSISTED BY THE FOLLOWING CONTRIBUTORS:
E. T. Balch, M.D., South Betid, Wash
Clarence Willard Butler, M. D.,
Montclair, N. J.
Prof. Edmund Carleton, M. D„ New
York.
Daniel W. Clausen, M. D., Philada.
Stuart Close, M. D., Brooklyn.
Edward Cranch, M. D., Erie, Pa.
W. S. Gee, M. D.. Hyde Park, Ill. . .
Clarence C. Howard, M. D., New York.
Samuel A. Kimb all, M. D., Boston.
Edmund J. Lee, M. D., Former Editor,
Philada.
A. McNeil, M. D , San Francisco.
C. F. Nichols. M. D., Boston.
Mahlon Preston, MD., Norristown. L?
George W. Shkrbino, M. D., Abilene, ’
- Texas.
C. Carleton Smith, M. D., Philada.
JVm Steinkauf, M. D., St. Charles, Mo.
PHILADELPHIA :
No. lies SPRUCE ST12.B3ET.
L O 3ST X> O TT
ALFRED HEATH & CO., Sole Agents for Great Britain,
114 Ebury Street, Eaton Square.
%&-'Xearly Subscription, $2.50. invariably in advance, Single numbers, 95 cts.
Ei tie eti at the Philaielplria Poet-Office as Second-Class Mail Matter.
				

